# Supplementary material for: Therapeutic Interventions for Music Performance Anxiety: A Systematic Review and Narrative Synthesis
Source: Behav Sci (Basel). 2025 Jan 26;15(2):138. doi: 10.3390/bs15020138 (PMC11851691; doi:10.3390/bs15020138)
Supplement: Supplementary file 1 [file behavsci-15-00138-s001.zip › behavsci-3348442-supplementary.pdf]

Supplementary Material Table S1. PRISMA Checklist

Section and Topic	Item #	Checklist item	Location where item is reported
**TITLE**			
Title	1	Identify the report as a systematic review.	Page 1
**ABSTRACT**			
Abstract	2	See the PRISMA 2020 for Abstracts checklist.	Page 1
**INTRODUCTION**			
Rationale	3	Describe the rationale for the review in the context of existing knowledge.	Pages 1-3
Objectives	4	Provide an explicit statement of the objective(s) or question(s) the review addresses.	Page 3
**METHODS**			
Eligibility criteria	5	Specify the inclusion and exclusion criteria for the review and how studies were grouped for the syntheses.	Pages 3-4
Information sources	6	Specify all databases, registers, websites, organisations, reference lists and other sources searched or consulted to identify studies. Specify the date when each source was last searched or consulted.	Page 3
Search strategy	7	Present the full search strategies for all databases, registers and websites, including any filters and limits used.	Pages 3-4
Selection process	8	Specify the methods used to decide whether a study met the inclusion criteria of the review, including how many reviewers screened each record and each report retrieved, whether they worked independently, and if applicable, details of automation tools used in the process.	Pages 3-4
Data collection process	9	Specify the methods used to collect data from reports, including how many reviewers collected data from each report, whether they worked independently, any processes for obtaining or confirming data from study investigators, and if applicable, details of automation tools used in the process.	Pages 3-4
Data items	10a	List and define all outcomes for which data were sought. Specify whether all results that were compatible with each outcome domain in each study were sought (e.g. for all measures, time points, analyses), and if not, the methods used to decide which results to collect.	Pages 4-6 and Tables 1 and 2
	10b	List and define all other variables for which data were sought (e.g. participant and intervention characteristics, funding sources). Describe any assumptions made about any missing or unclear information.	Pages 4-6 and Tables 1 and 2
Study risk of bias assessment	11	Specify the methods used to assess risk of bias in the included studies, including details of the tool(s) used, how many reviewers assessed each study and whether they worked independently, and if applicable, details of automation tools used in the process.	Pages 3-4 and Supplementary Materials Tables S2 and S3
Effect measures	12	Specify for each outcome the effect measure(s) (e.g. risk ratio, mean difference) used in the synthesis or presentation of results.	Table 1 indicates statistical significance for each study
Synthesis methods	13a	Describe the processes used to decide which studies were eligible for each synthesis (e.g. tabulating the study intervention characteristics and comparing against the planned groups for each synthesis (item #5)).	Pages 3-4 and Table 1
	13b	Describe any methods required to prepare the data for presentation or synthesis, such as handling of missing summary statistics, or data conversions.	Pages 3-4
	13c	Describe any methods used to tabulate or visually display results of individual studies and syntheses.	Pages 3-4
	13d	Describe any methods used to synthesize results and provide a rationale for the choice(s). If meta-analysis was performed, describe the model(s), method(s) to identify the presence and extent of statistical heterogeneity, and software package(s) used.	Pages 3-4 and Pages 26-27
	13e	Describe any methods used to explore possible causes of heterogeneity among study results (e.g. subgroup analysis, meta-regression).	Narrative synthesis, Pages 4 and Pages

Section and Topic	Item #	Checklist item	Location where item is reported
			22-23
	13f	Describe any sensitivity analyses conducted to assess robustness of the synthesized results.	N/A
Reporting bias assessment	14	Describe any methods used to assess risk of bias due to missing results in a synthesis (arising from reporting biases).	Pages 3-4 and Supplementary Materials Tables S2 and S3
Certainty assessment	15	Describe any methods used to assess certainty (or confidence) in the body of evidence for an outcome.	N/A
**RESULTS**			
Study selection	16a	Describe the results of the search and selection process, from the number of records identified in the search to the number of studies included in the review, ideally using a flow diagram.	Page 5 and Figure 1
	16b	Cite studies that might appear to meet the inclusion criteria, but which were excluded, and explain why they were excluded.	Figure 1
Study characteristics	17	Cite each included study and present its characteristics.	Pages 5-6 and Table 1
Risk of bias in studies	18	Present assessments of risk of bias for each included study.	Pages 5-6 and Supplementary Materials Tables S2 and S3
Results of individual studies	19	For all outcomes, present, for each study: (a) summary statistics for each group (where appropriate) and (b) an effect estimate and its precision (e.g. confidence/credible interval), ideally using structured tables or plots.	Table 1
Results of syntheses	20a	For each synthesis, briefly summarise the characteristics and risk of bias among contributing studies.	Pages 4-6, Table 2, and Pages 18-21
	20b	Present results of all statistical syntheses conducted. If meta-analysis was done, present for each the summary estimate and its precision (e.g. confidence/credible interval) and measures of statistical heterogeneity. If comparing groups, describe the direction of the effect.	N/A
	20c	Present results of all investigations of possible causes of heterogeneity among study results.	Pages 4-6, Table 2, and Pages 18-21
	20d	Present results of all sensitivity analyses conducted to assess the robustness of the synthesized results.	N/A
Reporting biases	21	Present assessments of risk of bias due to missing results (arising from reporting biases) for each synthesis assessed.	Pages 4-6 and Supplementary Materials Tables S2 and S3
Certainty of evidence	22	Present assessments of certainty (or confidence) in the body of evidence for each outcome assessed.	Table 2, Pages 18-21
**DISCUSSION**			
Discussion	23a	Provide a general interpretation of the results in the context of other evidence.	Pages 21-22 and Table 2
	23b	Discuss any limitations of the evidence included in the review.	Pages 22-23
	23c	Discuss any limitations of the review processes used.	Pages 22-23
	23d	Discuss implications of the results for practice, policy, and future research.	Page 23-24
**OTHER INFORMATION**			
Registration and protocol	24a	Provide registration information for the review, including register name and registration number, or state that the review was not registered.	Page 23

Section and Topic	Item #	Checklist item	Location where item is reported
	24b	Indicate where the review protocol can be accessed, or state that a protocol was not prepared.	N/A
	24c	Describe and explain any amendments to information provided at registration or in the protocol.	N/A
Support	25	Describe sources of financial or non-financial support for the review, and the role of the funders or sponsors in the review.	Page 24 and 25
Competing interests	26	Declare any competing interests of review authors.	Page 25
Availability of data, code and other materials	27	Report which of the following are publicly available and where they can be found: template data collection forms; data extracted from included studies; data used for all analyses; analytic code; any other materials used in the review.	N/A

Supplementary Material Table S2. JBI Critical Appraisal Tool Checklist for Quasi-Experimental Studies

Study	Is it clear in the study what is the "cause" and what is the "effect" (i.e. there is no confusion about which variable comes first)?	Was there a control group?	Were participants included in any comparisons similar?	Were the participants included in any comparisons receiving similar treatment/care, other than the exposure or intervention of interest?	Were there multiple measurements of the outcome, both pre and post the intervention/exposure ?	Were the outcomes of participants included in any comparisons measured in the same way?	Were outcomes measured in a reliable way?	Was follow-up complete and if not, were differences between groups in terms of their follow-up adequately described and analysed?	Was appropriate statistical analysis used?	Overall appraisal: Include or Exclude?
Bissonnette et al. (2015)	Yes	Yes	Yes	Yes	Yes	Yes	Yes	N/A - No follow-up.	Yes	Include
Blasco-Lafarga et al. (2022)	Yes	No	N/A	N/A	Yes	N/A	Yes	N/A - No follow-up.	Yes	Include
Braden et al. (2015)	Yes	Yes	Yes	Yes	Yes	Yes	Yes	Yes	Yes	Include
Brodsky and Sloboda (1997)	Yes	Yes	Yes	Yes	Yes	Yes	Yes	Yes	Yes	Include
Brooker (2018)	Yes	Yes	Yes	Yes	Yes	Yes	Yes	N/A - No follow-up	Yes	Include
Butzer et al. (2016)	Yes	Yes	Yes	Yes	Yes	Yes	Yes	N/A - No follow-up	Yes	Include
Chang et al. (2003)	Yes	Yes	Yes	Yes	Yes	Yes	Yes	N/A - No follow-up	Yes	Include
Choi et al. (2023)	Yes	No	N/A	N/A	Yes	N/A	Yes	N/A - No follow-up	Yes	Include
Clarke et al. (2020)	Yes	No	N/A	N/A	Yes	N/A	Yes	Yes	Yes	Include
Clark and Agras (1991)	Yes	Yes	Yes	Yes	Yes	Yes	Yes	Follow-up was incomplete. Differences between groups were adequately described but not analysed.	Yes	Include
Clements-Cortés et al. (2024)	Yes	No	N/A	N/A	Yes	N/A	Yes	N/A - No follow-up.	Yes	Include
Cohen and Bodner (2019)	Yes	Yes	Yes	Yes	Yes	Yes	Yes	N/A - No follow-up	Yes	Include

Esplen and Hodnett (1999)	Yes	No	N/A	N/A	Yes	N/A	Yes	N/A - No follow-up	Yes	Include
Gómez-López and Sánchez-Cabrero (2024)	Yes	No	N/A	N/A	Yes	N/A	Yes	Follow-up was incomplete. Differences between groups were adequately described but not analysed.	Yes	Include
Hoffman and Hanrahan (2014)	Yes	Yes	Yes	Yes	Yes	Yes	Yes	Yes	Yes	Include
Juncos et al. (2017)	Yes	No	N/A	N/A	Yes	N/A	Yes	Yes	Yes	Include
Kendrick et al. (1982)	Yes	Yes	Yes	Yes	Yes	Yes	Yes	Yes	Yes	Include
Kenny and Halls (2018)	Yes	Yes	Yes	No; although all participants completed the pedagogic skills session as a control condition, it was not neutral which could introduce confounders	Yes	Yes	Yes	Yes	Yes	Include
Khalsa et al. (2013)	Yes	Yes	Yes	Yes	Yes	Yes	Yes	N/A - No follow-up	Yes	Include
Kim (2005)	Yes	No, but participants did serve as their own controls	Yes	Yes	Yes	Yes	Yes	N/A - No follow-up	Yes	Include
Kim (2008)	Yes	No	Yes	Yes	Yes	Yes	Yes	N/A – No follow-up.	Yes	Include
Lin et al. (2008)	Yes	Yes	Yes	Yes	Yes	Yes	Yes	N/A - No follow-up.	Yes	Include
Mahony et al. (2022)	Yes	No	N/A	N/A	Yes	N/A	Yes	Yes	Yes	Include
Montello et al. (1990)	Yes	Yes	Yes	Yes	Yes	Yes	Yes	N/A - No follow-up.	Yes	Include
Moral-Bofill et al. (2022)	Yes	Yes	Yes	Yes	Yes	Yes	Yes	N/A - No follow-up.	Yes	Include
Nagel et al. (1989)	Yes	Yes	Yes	Yes	Yes	Yes	Yes	N/A - No follow-up.	Yes	Include
Osborne et al. (2007)	Yes	Yes	Yes	Yes	Yes	Yes	Yes	N/A - No follow-up.	Yes	Include
Reitman (2001)	Yes	Yes	Yes	Yes	Yes	Yes	Yes	N/A - No follow-up.	Yes	Include
Spahn et al. (2016)	Yes	Yes	Yes	Yes	Yes	Yes	Yes	N/A - No follow-up.	Yes	Include
Stanton (1994)	Yes	Yes	Yes	Yes	Yes	Yes	Yes	Unclear. Did not specify whether any participants were lost to follow-up.	Yes	Include

Stern et al. (2012)	Yes	No	N/A	N/A	Yes	N/A	Yes	Follow-up was incomplete. Differences between groups were adequately described and analysed.	Yes	Include
Steyn et al. (2016)	Yes	Yes	Yes	Yes	Yes	Yes	Yes	N/A - No follow-up.	Yes	Include
Su et al. (2010)	Yes	No	N/A	N/A	Yes	N/A	Yes	N/A - No follow-up.	Yes	Include
Sweeney and Horan (1982)	Yes	Yes	Yes	Yes	Yes	Yes	Yes	N/A - No follow-up.	Yes	Include
Tarrant and Leathem (2007)	Yes	No	N/A	N/A	Yes	N/A	Yes	Follow-up was incomplete. Differences between groups were adequately described but not analysed.	Yes	Include
Tief and Gröpel (2021)	Yes	Yes	Yes	No; the goal-setting control condition was not neutral which could introduce confounders	Yes	Yes	Yes	N/A - No follow-up.	Yes	Include
Thurber et al. (2010)	Yes	Yes	Yes	Yes	Yes	Yes	Yes	N/A - No follow-up.	Yes	Include
Valentine et al. (1995)	Yes	Yes	Yes	Yes	Yes	Yes	Yes	N/A - No follow-up.	Yes	Include

Supplementary Material Table S3. JBI Critical Appraisal Tool Checklist for Randomised Controlled Trials

**Study**	**Was true randomisation used for assignment of participants to treatment groups?**	**Was allocation to treatment groups concealed?**	**Were treatment groups similar at the baseline?**	**Were participants blind to treatment assignment?**	**Were those delivering the treatment blind to treatment assignment?**	**Were treatment groups treated identically other than the intervention of interest?**	**Were outcome assessors blind to treatment assignment?**	**Were outcomes measured in the same way for treatment groups?**	**Were outcomes measured in a reliable way?**	**Was follow-up complete and if not, were differences between groups in terms of their follow-up adequately described and analysed?**	**Were participants analysed in the groups to which they were randomised?**	**Was appropriate statistical analysis used?**	**Was the trial design appropriate and any deviations from the standard RCT design (individual randomisation, parallel groups) accounted for in the conduct and analysis of the trial?**	**Overall appraisal: Include or Exclude?**
Nwokenna et al. (2022)	Yes	Unclear; authors did not mention if the allocation was concealed	Yes	No; the nature of the intervention (music training) likely made	No; music educators were briefed and aware of group assignments	Yes	Unclear; authors did not mention whether assessors were blind to	Yes	Yes	Yes	Yes	Yes	Yes	Include

				blinding impractical			treatment assignments							
Wells et al. (2012)	Yes	Unclear; authors did not mention if the allocation was concealed	Yes	No; participants were aware that they were receiving biofeedback, slow breathing, or no treatment	Unclear; authors did not mention if the individuals delivering the interventions were blind to assignments	Yes	Unclear; authors did not mention whether assessors were blind to treatment assignments	Yes	Yes	Yes	Yes	Yes	Yes	Include
